# A vector whitefly endocytic receptor facilitates the entry of begomoviruses into its midgut cells via binding to virion capsid proteins

**DOI:** 10.1371/journal.ppat.1009053

**Published:** 2020-12-03

**Authors:** Jing Zhao, Teng Lei, Xin-Jia Zhang, Tian-Yan Yin, Xiao-Wei Wang, Shu-Sheng Liu

**Affiliations:** Ministry of Agriculture Key Laboratory of Molecular Biology of Crop Pathogens and Insects, Institute of Insect Sciences, Zhejiang University, Hangzhou, China; Agriculture and Agri-Food Canada, CANADA

## Abstract

Many circulative plant viruses transmitted by insect vectors are devastating to agriculture worldwide. The midgut wall of vector insects represents a major barrier and at the same time the key gate a circulative plant virus must cross for productive transmission. However, how these viruses enter insect midgut cells remains poorly understood. Here, we identified an endocytic receptor complex for begomoviruses in the midgut cells of their whitefly vector. Our results show that two whitefly proteins, BtCUBN and BtAMN, compose a receptor complex BtCubam, for which BtCUBN contributes a viral-binding region and BtAMN contributes to membrane anchorage. Begomoviruses appear to be internalized together with BtCubam via its interaction with the 12–19 CUB domains of BtCUBN via clathrin-dependent endocytosis. Functional analysis indicates that interruption of BtCUBN and BtAMN lead to reduction of virus acquisition and transmission by whitefly. In contrast, CUBN-begomovirus interaction was not observed in two non-competent whitefly-begomovirus combinations. These observations suggest a major role of the specific endocytic receptor in facilitating viral entry into vector midgut cells.

## Author summary

Many viruses depend on insect vectors for transmission and spread. Following ingestion by insect vectors, many viruses need to circulate in the vector via a sequential path of stylet-midgut-haemolymph-salivary glands and are finally inoculated into plants with saliva secretion. To complete this journey, virions have to cross many physical/physiological barriers, of which the insect midgut wall represents the first and one of the major challenges. While this route of virus circulation has been known for a long time, the physiological and molecular mechanisms underlying the crossing of these barriers by viruses are poorly understood. Working with begomoviruses, a group of plant viruses of economic significance worldwide, and their insect vectors, the whiteflies of the *Bemisia tabaci* complex, we found that upon virus infection, two vector proteins, cubilin (CUBN) and amnionless (AMN), form a virus receptor complex to uptake the virions and assist them to move through the apical membrane of whitefly midgut cells via clathrin-dependent endocytosis. These novel findings contribute to a better understanding on the molecular mechanisms of insect transmission of circulative viruses.

## Introduction

Plant viruses make up about half of the agents of emerging infectious plant diseases [1–3]. The majority of plant viruses are transmitted by hemipteran insects such as aphids, planthoppers, and whiteflies. Virus transmission by insect vectors can be non-circulative and circulative [4]. Non-circulative plant viruses are retained in the stylet, food canal or foregut of their insect vectors and then transmitted to plant hosts on which the vectors probe/feed [5–7]. Circulative plant viruses move through a sequential path of stylet-midgut-haemolymph-salivary glands in their vectors and are finally inoculated into plants with saliva secretion [8]. Accordingly, circulative viruses face four major challenges in movement within the vector: midgut invasion, midgut escape, salivary glands invasion and salivary glands escape [8–9]. Among these, midgut cells represent the first cellular barrier to the viruses. Initial encounters between virions and vector midgut cells are mediated through viral binding to cell surface molecules on the midgut apical membrane. Dissection of the cellular components involved in virus translocation from midgut lumen into midgut cells represents a critical step in advancing our understanding of virus-vector interactions.

Endocytosis is widely involved in the entry of viruses into vector cells. The brush border and microvilli at the apical membrane of midgut epithelial cells provide an ideal site for viral endocytosis [10–12]. For endocytosis to occur, receptors are required for the recruitment of viruses into endocytic structures such as clathrin-coated pits [12–13]. Many vector or host receptors of animal viruses have been reported [12,14]. However, for plant viruses, only two such protein receptors have been reported: the membrane alanyl aminopeptidase N as a gut receptor in an aphid vector for *Pea enation mosaic virus* [15–16], and the sugar transporter 6 of a planthopper vector for the entry of *Rice stripe virus* into midgut epithelial cells [17].

Begomoviruses (family *Geminiviridae*, genus *Begomovirus*) are causative agents of many serious viral diseases of crops [18–19], and are transmitted by whiteflies of the *Bemisia tabaci* cryptic species complex in a circulative manner [20–21]. Tomato yellow leaf curl virus (TYLCV), which is listed in the top 10 plant viruses based on economic damage, can be transmitted by a number of species of the whitefly complex such as Middle East Asia Minor 1 (MEAM1) [2,22–24]. Our previous work showed that TYLCV may exploit clathrin-dependent endocytosis to enter the MEAM1 whitefly midgut cells [25]. And the presence of intracellular vesicle structures in which TYLCV accumulates was verified in the midgut cells of the MEAM1 whitefly [26–27]. However, the membrane receptors required for viral endocytosis are hitherto unknown.

Begomovirus coat protein (CP) is the only known structural protein involved in its movement in insect vectors [28]. In this study, we used GST pull-down followed by LC-MS/MS analysis to screen the binding partners of TYLCV CP in the MEAM1 whitefly, and selected one protein, namely cubilin (BtCUBN), for further analysis as mammal CUBN serves as a classical endocytic receptor for many ligands [29]. Using a series of molecular techniques including gene transcription analysis, immunogold electron microscopy (IEM), immunofluorescence, co-immunoprecipitation (Co-IP) assay, and virus acquisition and transmission trails, we found that BtCUBN and amnionless (BtAMN) formed a BtCubam receptor complex that mediates the entry of begomoviruses into vector midgut cells in a clathrin-dependent manner.

## Results

### BtCUBN is a potential endocytic receptor in the MEAM1 whitefly midgut cells

**Fig 1:**
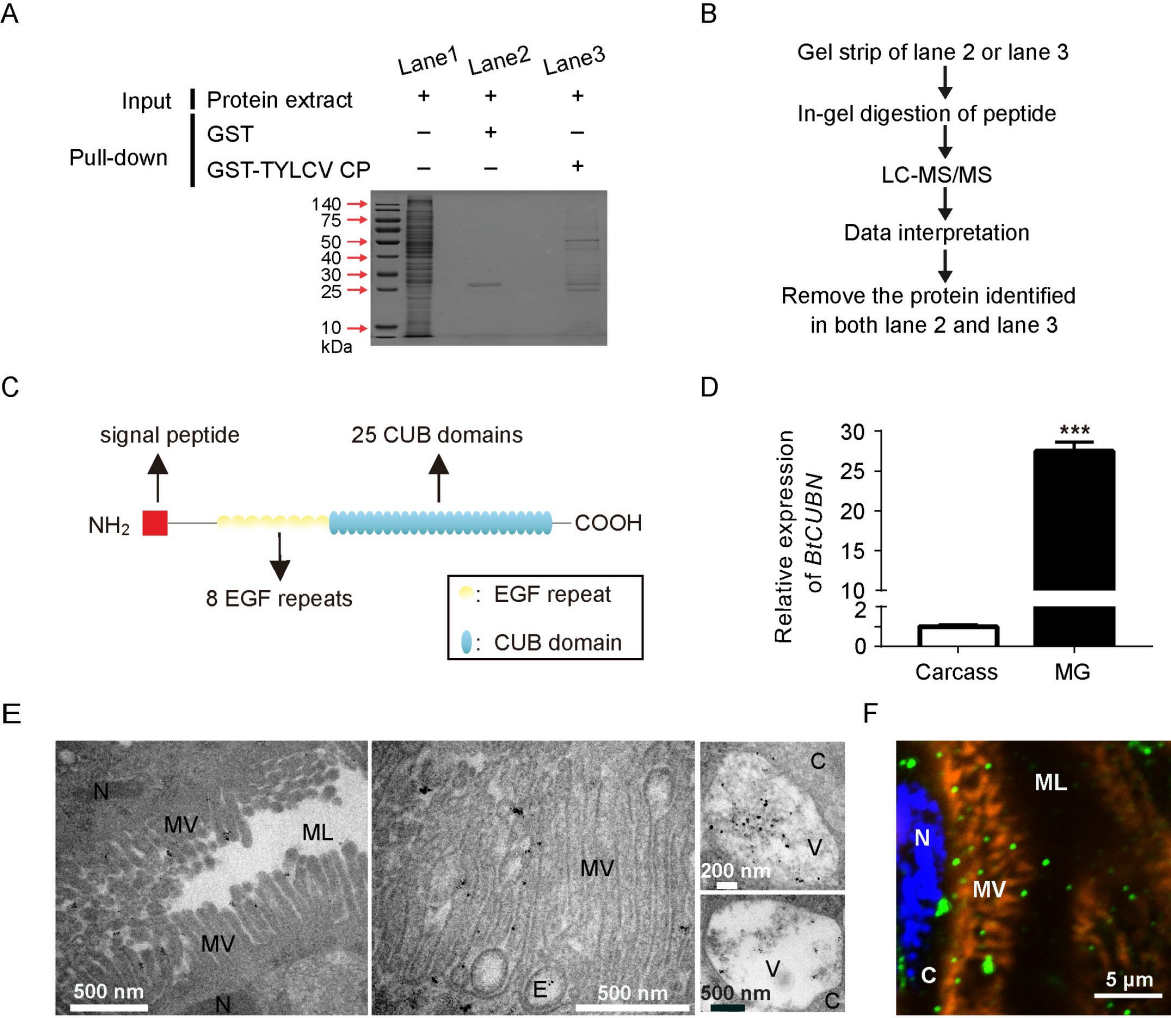
BtCUBN captured by GST pull-down coupled with LC-MS/MS. (A) GST pull-down analysis using GST-TYLCV CP and protein extract of the MEAM1 whitefly, and coomassie brilliant blue was used to stain gel to visualize proteins. (B) Workflow of LC/MS-MS and data analysis to identify the binding partners of TYLCV CP in the MEAM1 whitefly. (C) Schematic representation of BtCUBN structure. (D) *BtCUBN* transcription levels in the MEAM1 whitefly midgut and carcass. Midguts of female adults were removed and the remains were collected as carcass. Data were presented as mean ± SEM, n = 3 (Independent t-test, ***P < 0.001). (E-F) Immunogold (≈10 nm gold, E) or immunofluorescence (F) detections of BtCUBN distribution in the MEAM1 whitefly midgut cells by its rabbit mAb. The first two pictures of IEM displayed the localization of BtCUBN in the midgut microvill (MV), and the third picture of IEM showed the localization of BtCUBN in the cytoplasm (C) of midgut cell. the MEAM1BtCUBN was labelled with Alexa Fluor 647 (green); midgut apical membrane was stained by phalloidine conjugated to Alexa Fluor 488 (orange); cell nucleus was stained by DAPI (Alexa Fluor 405, blue). ML: midgut lumen; MV: microvilli; C: cytoplasm; N: nucleus; V: vesicle; BM: basal membrane.

We conducted GST pull-down (Fig 1A) coupled with LC-MS/MS assay (Fig 1B) to identify the putative binding partners of TYLCV CP in the MEAM1 whitefly. In total, 44 proteins of the MEAM1 whitefly, for each of which the number of unique peptides is ≥ 2, were identified (S1 Table). Among them, BtCUBN (≈ 423 kDa; NCBI accession number: XP_018908774.1), previously known to be involved in the process of receptor-mediated endocytosis (GO:0006898) with receptor activity (GO:0004872), was subjected to further investigation. Sequence analysis revealed that BtCUBN shares 29% amino acid identity with human CUBN, and is similar in structure to human CUBN which contains 8 EGF (epidermal growth factor) repeats and 27 CUB (complement subcomponents C1r/C1s, EGF-related sea urchin protein and bone morphogenic protein-1) domains. BtCUBN contains 8 EGF repeats and 25 CUB domains (Fig 1C). Compared with that in the MEAM1 whitefly carcass (whole body sans-gut), *BtCUBN* exhibited a much higher transcription level in the midgut (Fig 1D). As a candidate receptor, BtCUBN was detected at the microvilli (MV) or cytoplasm in whitefly midgut cells (Fig 1E and 1F), and moreover in intracellular vesicle structures (Fig 1E).

### *In vivo* and *in vitro* interactions between TYLCV CP and BtCUBN

**Fig 2:**
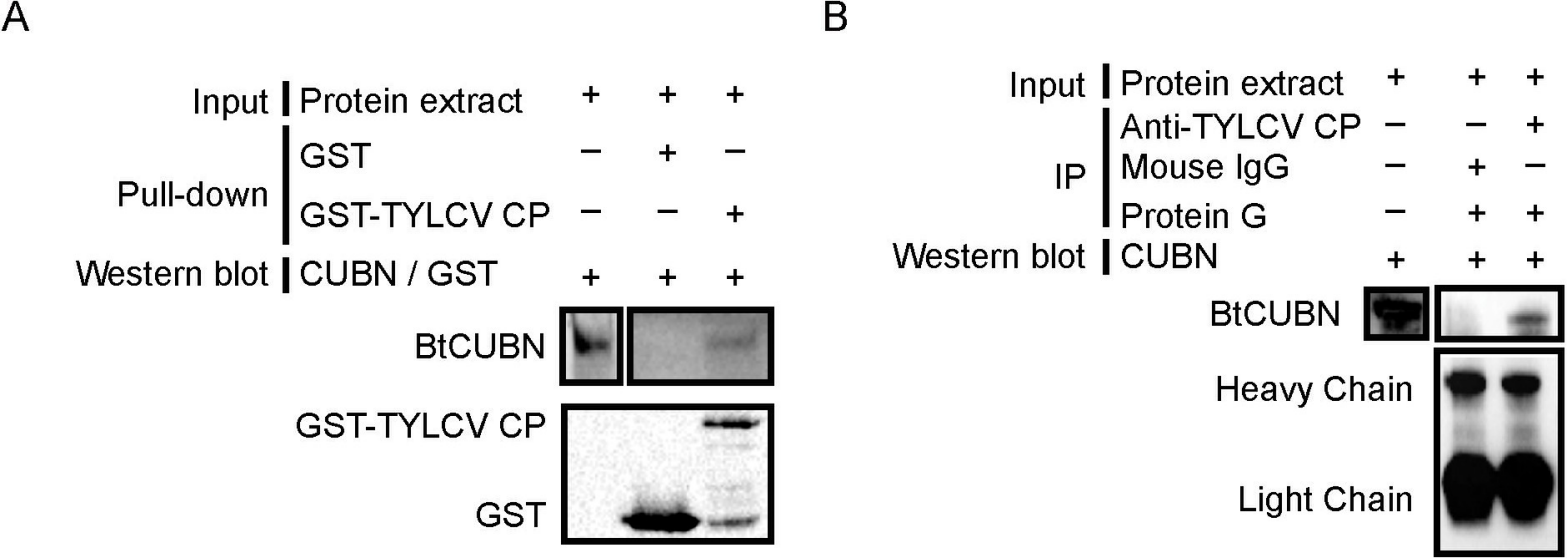
Verification of interaction between BtCUBN and TYLCV CP. (A) GST pull-down analysis. Native proteins of the MEAM1 whitefly were extracted by cytoplasmic extraction buffer (Cat. No. SC-003; Invent), which was used as prey protein; GST or GST-TYLCV CP was used as bait protein. After GST pull-down assay, anti-CUBN rabbit mAb was used to detect prey protein; anti-GST mouse mAb was used to detect bait protein. (B) Co-IP analysis. After a 7 d virus acquisition, native proteins of the MEAM1 whitefly were extracted by cytoplasmic extraction buffer (Cat. No. SC-003; Invent), and then incubated with anti-TYLCV CP mouse mAb (corresponding pre-immune sera was used as control) and protein G-Sepharose beads. Anti-CUBN rabbit mAb was used to detect BtCUBN. BtCUBN, ≈ 423 kDa; GST, ≈ 26 kDa; GST-TYLCV CP, ≈ 54 kDa; light chain, ≈ 26 kDa; heavy chain, ≈ 55 kDa.

**Fig 3:**
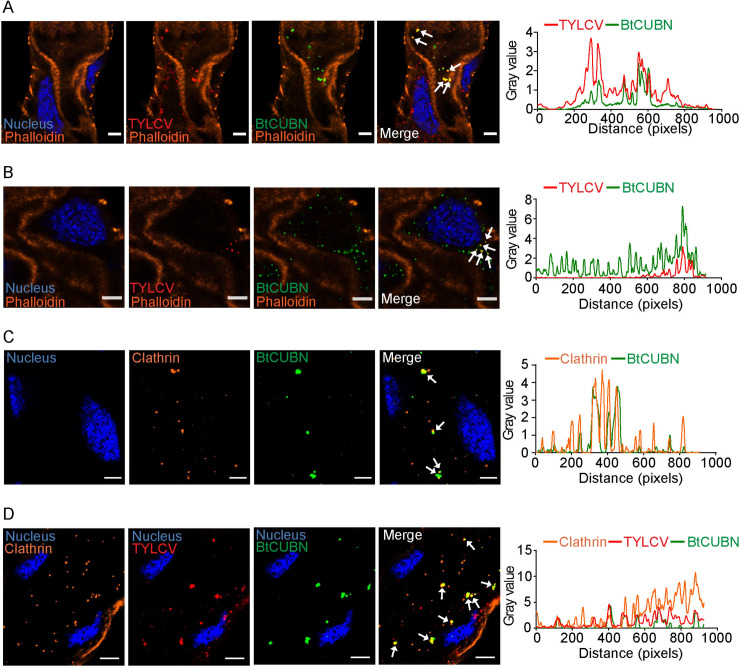
TYLCV internalized with BtCUBN via clathrin-dependent endocytosis. The MEAM1 adults were allowed to feed on TYLCV-infected plants for a 48 h AAP for immunofluorescence analysis. (A-B) Co-localization between extracellular (A) or intracellular (B) TYLCV CP and BtCUBN in the MEAM1 whitefly midgut. (C) Co-localization between BtCUBN and clathrin. (H) Co-localization among TYLCV CP, BtCUBN and clathrin. In A-B, BtCUBN was labelled by Alex Flour 647 (green), midgut apical membrane was stained by phalloidine conjugated to Alex Flour 488 (orange), TYLCV CP was detected by its mouse mAb (Alex Flour 549, red) and cell nucleus was stained by DAPI (Alex Flour 405, blue). In C-D, BtCUBN was labelled by Alex Flour 488 (green), clathrin was labelled with Alex Flour 549 (orange), TYLCV CP was labelled with Alex Flour 647 (red) and cell nucleus was stained by DAPI (Alex Flour 405, blue). Scale bar, 5μm. The plot profile presented was used to help verify the visual co-localization.

To further examine the interaction between BtCUBN and TYLCV CP, we conducted GST pull-down (Fig 2A) and Co-IP (Fig 2B). *In vitro* GST pull-down assay showed that BtCUBN could be co-eluted with GST-TYLCV CP (Fig 2A), and anti-TYLCV CP mouse mAb antibodies captured the interaction between BtCUBN and TYLCV CP *in vivo* (Fig 2B). We further conducted immunofluorescence to examine their co-localization in the midgut of TYLCV-infected MEAM1 whiteflies. Co-localization of extracellular virions with BtCUBN was clearly observed on the side of midgut microvilli (Fig 3A), and intracellular virions also co-localized with BtCUBN inside midgut cells (Fig 3B). As TYLCV is known to move from outside to inside whitefly midgut cells (S1A and S1B Fig), these observations suggest that viruses may be internalized together with BtCUBN.

### Internalization of TYLCV with BtCUBN via clathrin-dependent endocytosis

CUBN has been reported to act as an endocytic receptor in opossum kidney epithelial cells via a clathrin-dependent manner for the internalization of fluorescein isothiocyanate-labelled human immunoglobulin G [30]. Here, we conducted immunofluorescence assays to explore whether the internalization of TYLCV with BtCUBN occurred in a clathrin-dependent manner. A clear co-localization of BtCUBN with clathrin suggests the endocytosis of BtCUBN may be clathrin-dependent (Fig 3C). Further, the tripartite co-localization of BtCUBN, TYLCV and clathrin (Fig 3D) suggests that TYLCV and BtCUBN may be internalized via clathrin-dependent endocytosis.

### Differential intracellular trafficking routes of TYLCV and BtCUBN

**Fig 4:**
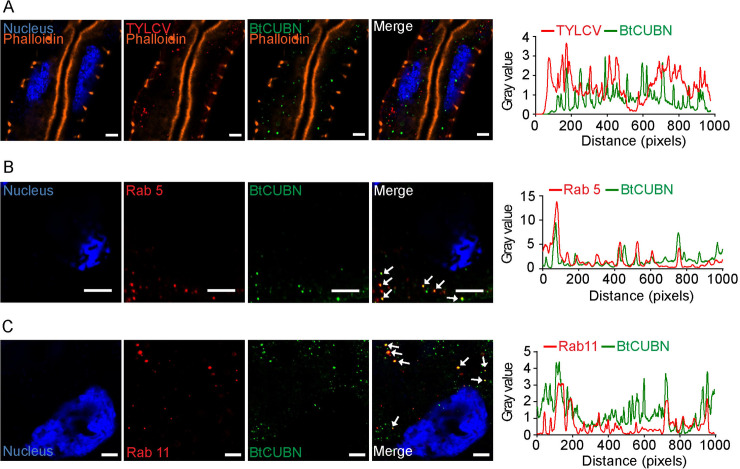
Co-localization among TYLCV CP, BtCUBN and endosomes. The MEAM1 adults were allowed to feed on TYLCV-infected plants for a 48 h AAP for immunofluorescence analysis. (A) Dissociation between intracellular TYLCV and BtCUBN. Viruses were detected by Alex Flour 549 (red); BtCUBN was labelled by its rabbit mAb (Alex Flour 647, green); midgut apical membrane was stained by phalloidine (Alex Flour 488, orange); cell nucleus was stained by DAPI (Alex Flour 405, blue). Scale bar, 5μm. (B) Co-localization between BtCUBN and Rab 5. (C) Co-localization between BtCUBN and Rab11. In B-C, antibodies against Rab5 and Rab11 were used to label early endosomes and recycling endosomes respectively (Alex Flour 647, red), BtCUBN was labelled by anti-BtCUBN_3593-3847aa_ mouse pAb antibody with Alex Flour 549 (green), cell nucleus was stained by DAPI (Alex Flour 405, blue). Scale bar, 5μm. The plot profile presented was used to verify the visual co-localization.

After entry into midgut cells, begomoviruses will move into vector haemolymph [11,31–32]. Cellular receptors involved in transmembrane transport, such as BtCUBN, are supposed to return to cell surface [33]. In this study, we found that, intracellular viruses did not always co-localize with BtCUBN. In some cases, virions did not co-localize with BtCUBN (Fig 4A). According to Xia et al. [27], the trafficking of TYLCV within the MEAM1 whitefly midgut cells relies on Rab5-labeled early endosome, but is unrelated to Rab11-labeled recycling endosomes. Here, we speculated that, BtCUBN may experience a differential endosomal trafficking with TYLCV. We found that BtCUBN co-localized not only with Rab5-labeled early endosomes (Fig 4B), but also with Rab11-labeled recycling endosomes (Fig 4C). In theory, the low pH in early endosome renders it an ideal location for the dissociation of receptor-ligand complex [34]. We thus suggest that BtCUBN may dissociate from TYLCV in early endosomes and then return to the surface of midgut cells via recycling endosomes.

### BtCUBN forms a receptor complex with BtAMN *in vivo*

**Fig 5:**
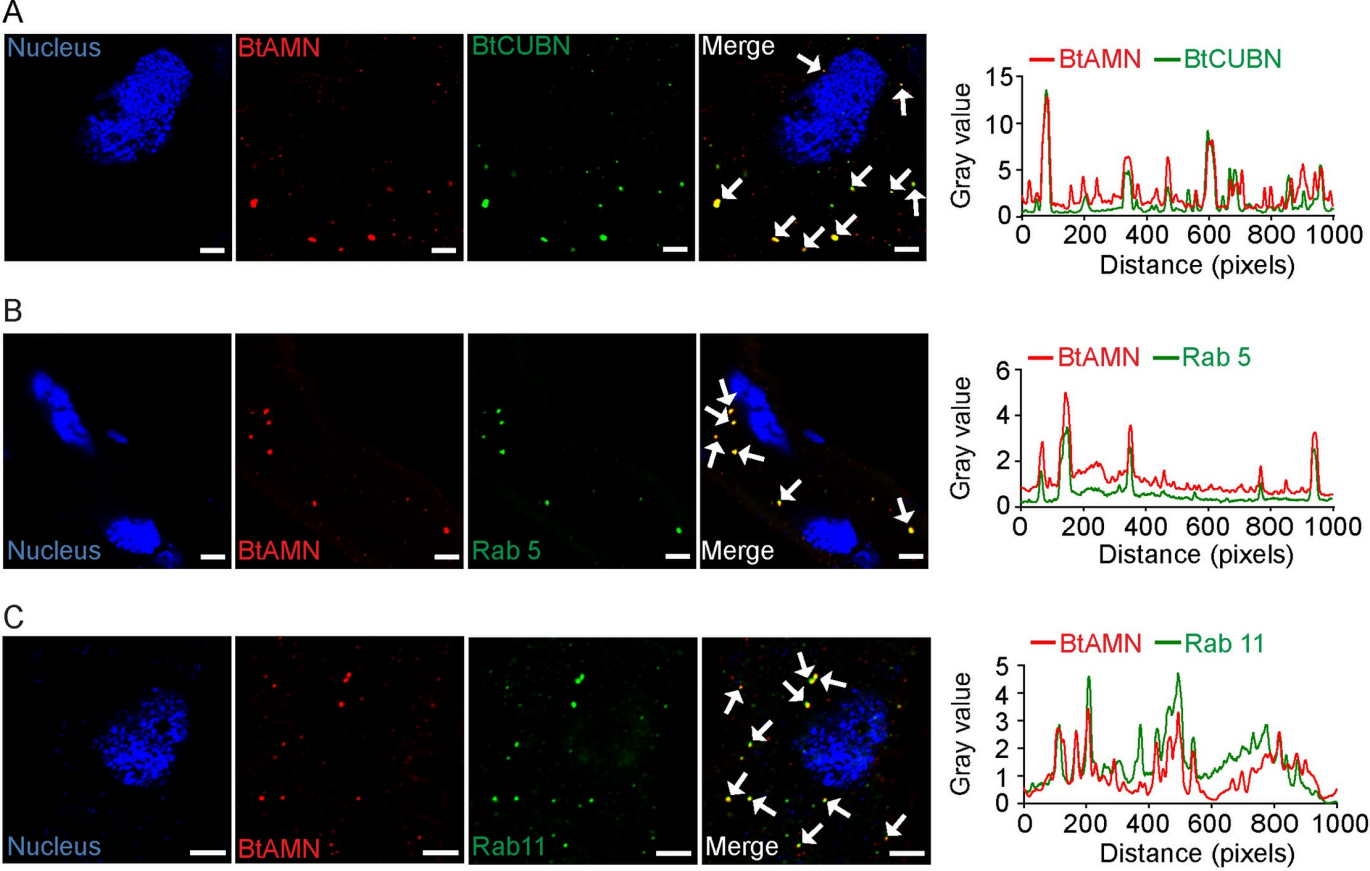
Co-localization between BtAMN and BtCUBN or endosomes. The MEAM1 adults were allowed to feed on TYLCV-infected plants for a 48 h AAP for immunofluorescence analysis. (A) Co-localization between BtAMN and BtCUBN in the MEAM1 whitefly midgut cells. BtAMN was labelled with Alex Flour 549 (red); midgut apical membrane was stained by phalloidine conjugated to Alex Flour 488 (orange); cell nucleus was stained by DAPI (Alex Flour 405, blue); anti-CUBN rabbit mAb was labelled with Alex Flour 647 (green). Scale bar, 5μm. (B-C) Co-localization between BtAMN and endosomes: (B) Rab 5-labelled early endosome; (C) Rab 11-labelled recycling endosome. Antibodies against Rab5 and Rab11 were used to label early endosomes and recycling endosomes respectively (Alex Flour 647, green), BtAMN was labelled with Alex Flour 549 (red) and cell nucleus was stained by DAPI (Alex Flour 405, blue). Scale bar, 5μm. The plot profile presented was used to help verify the visual co-localization.

Due to the lack of a transmembrane region and glycosylphosphatidylinositol anchorin, mammalian or *Drosophila* CUBN forms a functional receptor complex (designated as Cubam) with a transmembrane protein AMN, which is essential for the membrane targeting and endocytosis of CUBN with its ligand [33,35–40]. Analogously, no transmembrane region was found in BtCUBN (Fig 1C). Therefore, we searched the MEAM1 whitefly genome and identified BtAMN (≈ 54 kDa; NCBI accession number: XP_018900553.1). BtAMN contains a signal peptide and a transmembrane domain (S2A Fig). Similar to BtCUBN, the transcription of *BtAMN* in the MEAM1 whitefly midgut was significantly higher than that in carcass (S2B Fig); and the presence of BtAMN in the MEAM1 whitefly midgut cells was also detected in microvilli and cytoplasm (S2C Fig). Then, we conducted immunofluorescence assay and detected clear co-localization of BtCUBN with BtAMN *in vivo* (Fig 5A). Intracellular endosomal trafficking analysis demonstrated that BtAMN co-localized not only with Rab5-labeled early endosomes (Fig 5B), but also with Rab11-labeled recycling endosome (Fig 5C), indicating that BtAMN shares the same intracellular trafficking route with BtCUBN. These data suggest that BtCUBN and BtAMN may form a functional complex in the MEAM1 whitefly midgut.

### TYLCV CP binds to BtCubam receptor complex via its interaction with the 12–19 CUB domains of BtCUBN

**Fig 6:**
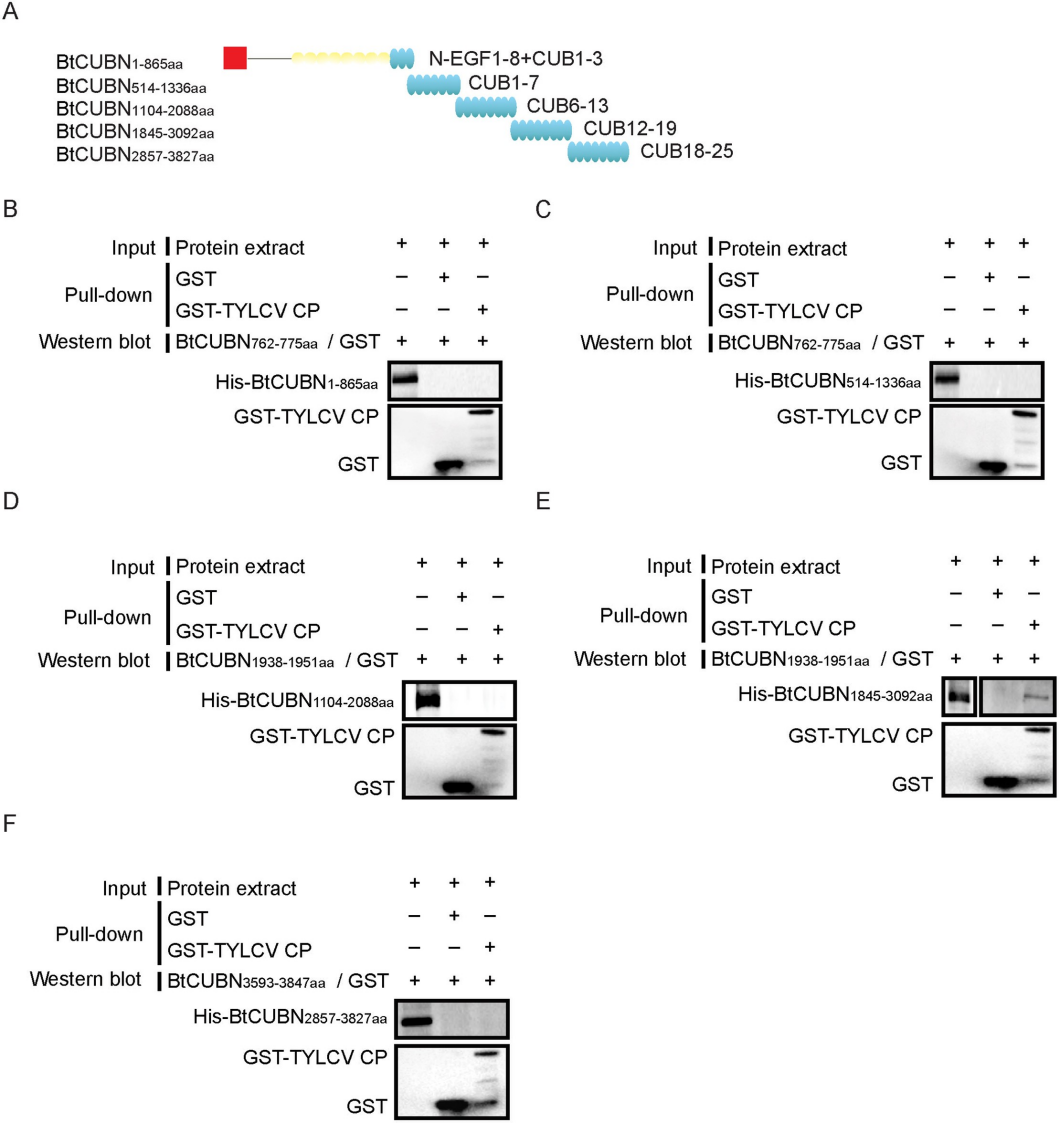
TYLCV binds to the 12–19 CUB domains of BtCUBN. (A) Schematic representation of five overlapping regions of BtCUBN. (B-F) The interaction between TYLCV CP and different regions of BtCUBN was analyzed using GST pull-down assay. Purified GST or GST-TYLCV CP was used as bait protein, and these five overlapping regions of BtCUBN were individually expressed in Sf 9 cells as His-tag fusion proteins (prey proteins): (B) BtCUBN_1-865aa_ (1–8 N-EGF 1–8+ 1–3 CUB domains of BtCUBN), ≈ 95 kDa; (C) BtCUBN_514-1336aa_ (1–7 CUB domains of BtCUBN), ≈ 91 kDa; (D) BtCUBN_1104-2088aa_ (6–13 CUB domains of BtCUBN), ≈ 140 kDa; (E) BtCUBN_1845-3092aa_ (12–19 CUB domains of BtCUBN), ≈ 137 kDa; (F) BtCUBN_2857-3827aa_ (18–25 CUB domains of BtCUBN), ≈ 106 kDa. For B-C, anti-BtCUBN_762-775aa_ rabbit pAb was used to detect prey protein; for D-E, anti-BtCUBN_1938-1955aa_ rabbit pAb was used to detect prey protein; for F, anti-BtCUBN_3593-3847aa_ mouse pAb was used to detect prey protein. Anti-GST mouse mAb was used to detect bait protein. GST, ≈ 26 kDa; GST-TYLCV CP, ≈ 54 kDa.

In mammals, the binding sites of some ligands in Cubam receptor complex have been detected. For example, the intrinsic factor-cobalamin complex and albumin were reported to bind to the 113-residue amino terminus that includes the EGF repeats and 6–8 CUB domains [41]; protein RAP was reported to bind to 13–14 CUB domains [42]. To explore the binding sites of TYLCV CP in the BtCubam receptor complex, we firstly generated HA-BtAMN or His-BtAMN fusion protein in Sf 9 cells, and found that both His-BtAMN (S3A and S3B Fig) and HA-BtAMN (S3C and S3D Fig) were not co-eluted with GST-TYLCV CP using GST pull-down assay, indicating that TYLCV CP did not bind to BtAMN directly. Then, we conducted multiple GST pull-down analyses to determine the viral binding sites in the BtCubam complex. Five overlapping regions of BtCUBN (Fig 6A) were individually expressed in Sf 9 cells as His-tag fusion proteins. Afterwards, we performed GST pull-down analysis to investigate the interaction between TYLCV CP and each of these fragments. GST-TYLCV CP exhibited a binding activity only to the 12–19 CUB domains of BtCUBN (Fig 6E), and the other four regions were not captured as binding partner of GST-TYLCV CP (Fig 6B, 6C, 6D and 6F). These data suggest that the binding sites of TYLCV CP in BtCubam receptor complex may be located in the 12–19 CUB domains of BtCUBN.

### The roles of BtCUBN and BtAMN in TYLCV acquisition and transmission

**Fig 7:**
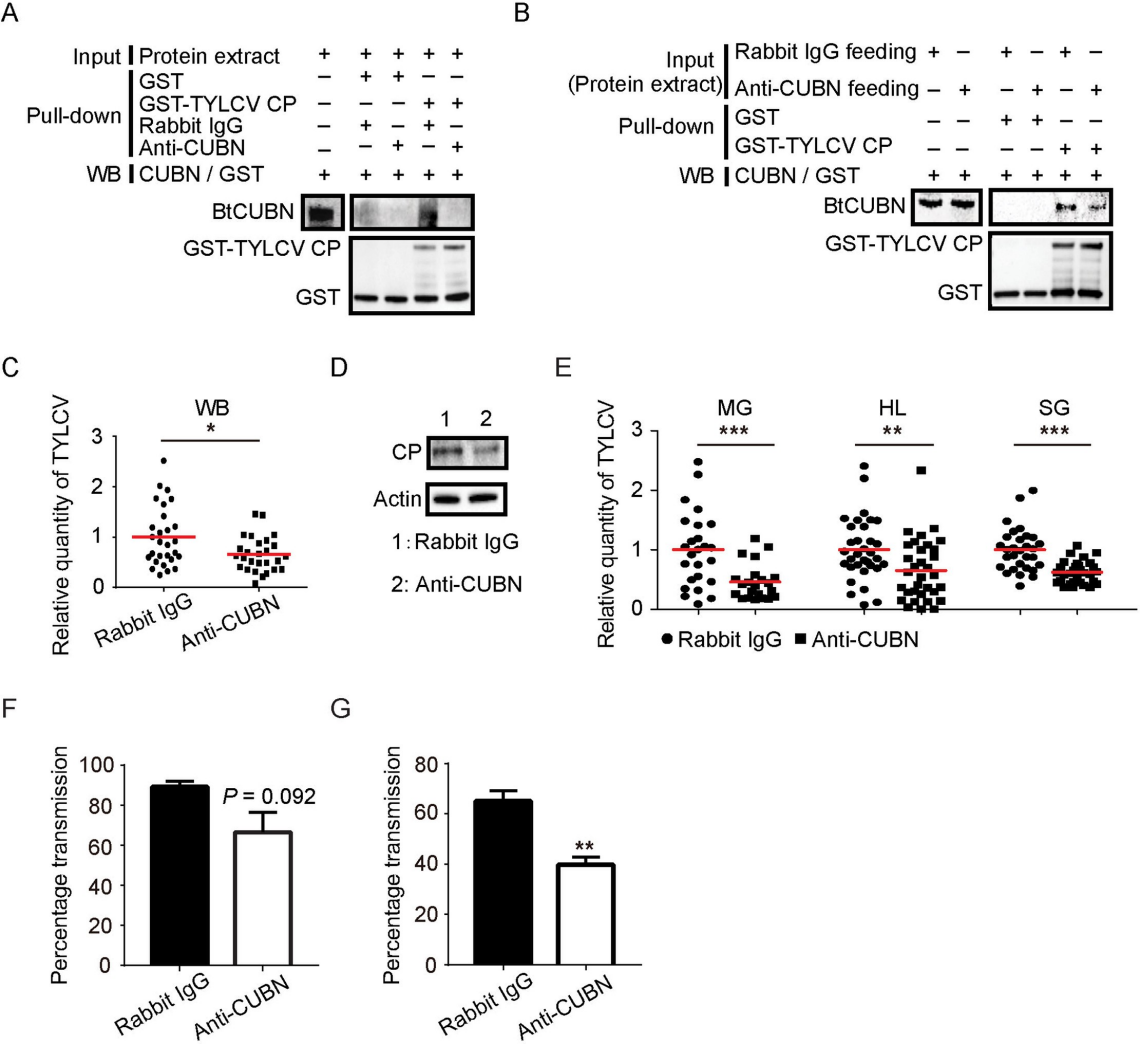
Disruption of the interaction between BtCUBN and TYLCV CP leads to impaired virus acquisition and transmission. (A) Anti-CUBN rabbit mAb blocks the interaction between BtCUBN and TYLCV CP. (B) Anti-CUBN rabbit mAb-feeding treatment reduces the interaction between BtCUBN and TYLCV CP as shown by GST pull-down. (C-F) TYLCV level in the MEAM1 whitefly whole body was analyzed by qPCR (C) or western blot (D) respectively; relative viral amounts in the MEAM1 whitefly different tissues were analyzed by qPCR (E); two females were collected and virus transmission was performed (F); data shown are mean ± SEM, n = 3. (G) Viral transmission efficiency following membrane feeding of anti-CUBN rabbit mAb and TYLCV virions, six females were collected for virus transmission, data shown are mean ± SEM, n = 3. WB: whole body; MG: midgut; HL: haemolymph; SG: salivary gland. Statistically significant differences are indicated as: *P < 0.05; **P < 0.01; ***P < 0.001 (Independent t-test for C, E and F). BtCUBN, ≈ 423 kDa; GST, ≈ 26 kDa; GST-TYLCV CP, ≈ 54 kDa; light chain, ≈ 26 kDa; heavy chain, ≈ 55kDa; TYLCV CP, ≈ 28 kDa; Actin, ≈ 42 kDa.

**Fig 8:**
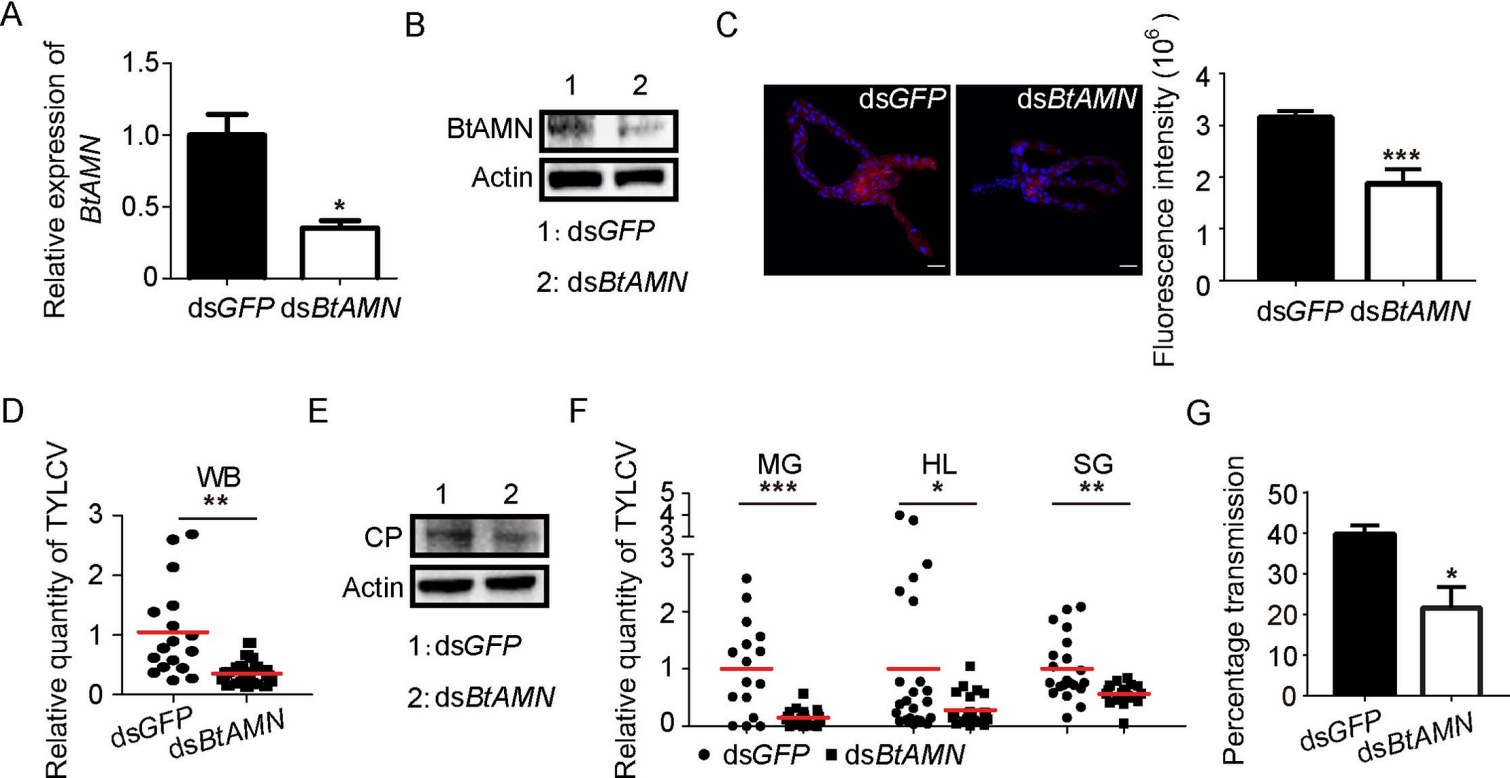
Effect of ds*BtAMN* interference on viral acquisition and transmission. (A) Relative gene transcription level of *BtAMN* after dsRNA micro-injection. ds*GFP* was used as control; data shown are mean ± SEM, n = 3. (B) Protein expression level of BtAMN after dsRNA micro-injection. β-Actin was used as loading control. (C) Detection of BtAMN in the midgut of ds*GFP* or ds*BtAMN*-treated the MEAM1 whitefly. BtAMN was detected with Alex Flour 549 (red); nucleus was stained with DAPI (Alex Flour 405, blue). Six-eight midguts were dissected for fluorescence intensities analysis; data were presented as mean ± SEM. Representative images that were used to generate the data here are provided in S9 Fig. (D-G) After ds*BtAMN* interference and 24 h virus acquisition, TYLCV level in the MEAM1 whitefly whole body was analyzed by qPCR (D) or western blot (E) respectively; relative viral amount in the MEAM1 whitefly different tissues were analyzed by qPCR (F); two females were collected and virus transmission was performed (G); data shown are mean ± SEM, n = 3. WB: whole body; MG: midgut; HL: haemolymph; SG: salivary gland. Statistically significant differences are indicated as: *, P < 0.05; **, P < 0.01; ***, P < 0.001 (Independent t-test for A, C, D, F and G). BtAMN, ≈ 54 kDa; TYLCV CP, ≈ 28 kDa; Actin, ≈ 42 kDa.

Since BtCUBN serves as viral binding sites, anti-CUBN rabbit mAb was used to interfere with the interaction between BtCUBN and TYLCV CP. GST pull-down assay showed that the anti-CUBN rabbit mAb treatment *in vitro* abolished the interaction between BtCUBN and TYLCV CP (Fig 7A). In addition, membrane feeding of anti-CUBN rabbit mAb weakened the binding of BtCUBN to GST-TYLCV CP (Fig 7B). Following antibody blocking and subsequent viral acquisition, qPCR and western blot analysis showed that anti-CUBN rabbit mAb blocking decreased the level of TYLCV CP in the MEAM1 whitefly (Fig 7C and 7D). qPCR data likewise demonstrated significant reductions of relative viral quantity in the midgut, haemolymph and salivary gland of the MEAM1 whitefly (Fig 7E). When a `virus acquisition test was performed post antibody treatment, administration of anti-CUBN rabbit mAb in the MEAM1 whitefly seemed to decrease begomovirus transmission efficiency but no statistical significance was found (Fig 7F). However, when the MEAM1 whiteflies were allowed to acquire anti-CUBN rabbit mAb and virions at the same time using membrane feeding, a significant reduction in viral transmission efficiency was observed (Fig 7G). In addition, since the expression of BtAMN is required for the membrane targeting of BtCUBN, we used ds*BtAMN* to interfere with the expression of BtAMN to explore its role in viral acquisition and transmission by whitefly. Compared to *dsGFP* control, qPCR, western blot and immunofluorescence demonstrated a lower expression of BtAMN post ds*BtAMN* treatment (Fig 8A, 8B and 8C). Following ds*BtAMN* interference and subsequent viral acquisition, both the viral quantities in the whole body (Fig 8D and 8E) and organs (Fig 8F) of the MEAM1 whitefly, as well as the viral transmission efficiency by whitefly (Fig 8G), were significantly reduced. These data indicate that both BtCUBN and BtAMN play important role in the acquisition and transmission of TYLCV by the MEAM1 whitefly.

### BtCubam is involved in the transport of tomato yellow leaf curl China virus in the MEAM1 whitefly

To explore whether BtCubam plays a conserved role in the MEAM1 whitefly-begomovirus interactions, we conducted similar tests with another begomovirus, tomato yellow leaf curl China virus (TYLCCNV), which is likewise efficiently transmitted by the MEAM1 whitefly [43–44]. Immunofluorescence assay showed a clear tripartite co-localization among TYLCCNV, BtCUBN and clathrin (S4A Fig). Next, antibody feeding or dsRNA micro-injection and subsequent virus acquisition and transmission trails were performed. Administration of anti-CUBN rabbit mAb or ds*BtAMN* significantly reduced the quantity of virus in the MEAM1 whitefly whole body and organs S4B and S4C Fig) and viral transmission efficiency by the MEAM1 whitefly (S4D and S4E Fig). These data indicate BtCubam receptor complex may play a conserved role in whitefly-begomovirus interactions.

### CLCuMuV CP exhibits no binding capacity to BtCUBN

Cotton leaf curl Multan virus (CLCuMuV), one of the major begomoviruses associated with cotton leaf curl disease, could be transmitted by the Asia II 1 species of *B*. *tabaci* whitefly complex with high efficiency, but not by the MEAM1 whitefly when using cotton as the host [45]. This differential transmission was considered to be associated with the inability of CLCuMuV to cross the vector midgut, as no virions were detected in the haemolymph of the MEAM1 whitefly [45]. Here, we used GST-CLCuMuV CP as bait protein to determine if it could bind to BtCUBN, and the data of GST pull-down demonstrated no binding activity between the two proteins (S5A Fig). Using co-IP analysis with anti-TYLCV CP mouse mAb antibody, we did not detect the interaction between BtCUBN and CLCuMuV CP *in vivo* either (S5B Fig). We detected very weak viral signals in the midgut of viruliferous MEAM1 whitefly adults that had fed on CLCuMuV-infected plants for 7 days (S5C Fig). Further analysis showed no co-localization between CLCuMuV CP and BtCUBN (S5D Fig). These findings indicate that BtCUBN **exhibits** no binding activity to CLCuMuV CP.

### TYLCV CP exhibits no binding capacity to TvCUBN

The greenhouse whitefly *Trialeurodes vaporariorum* does not transmit begomoviruses due to the inability of begomoviruses to cross the selective midgut invading barrier; viruses are restricted in the midgut lumen and no viruses were observed inside midgut cells [11,26,46]. We searched for TvCUBN in the transcriptome of the greenhouse whitefly (PRJNA541208). GST pull-down and co-IP analysis were conducted to examine the interaction of TvCUBN with TYLCV CP, and no interaction between TvCUBN and TYLCV CP was identified (S6A and S6B Fig). The structure of the greenhouse whitefly midgut is similar to that of the MEAM1 whitefly (S6C Fig). Consistent with a previous study [46], immunofluorescence of virions showed that viral signals were found only in the midgut lumen of the TYLCV-infected greenhouse whitefly (S6D Fig). Further immunofluorescence analysis showed no co-localization between TYLCV CP and TvCUBN in the greenhouse whitefly midgut (S6E Fig). These findings indicate that TvCUBN exhibits no binding capacity to TYLCV CP.

## Discussion

**Fig 9:**
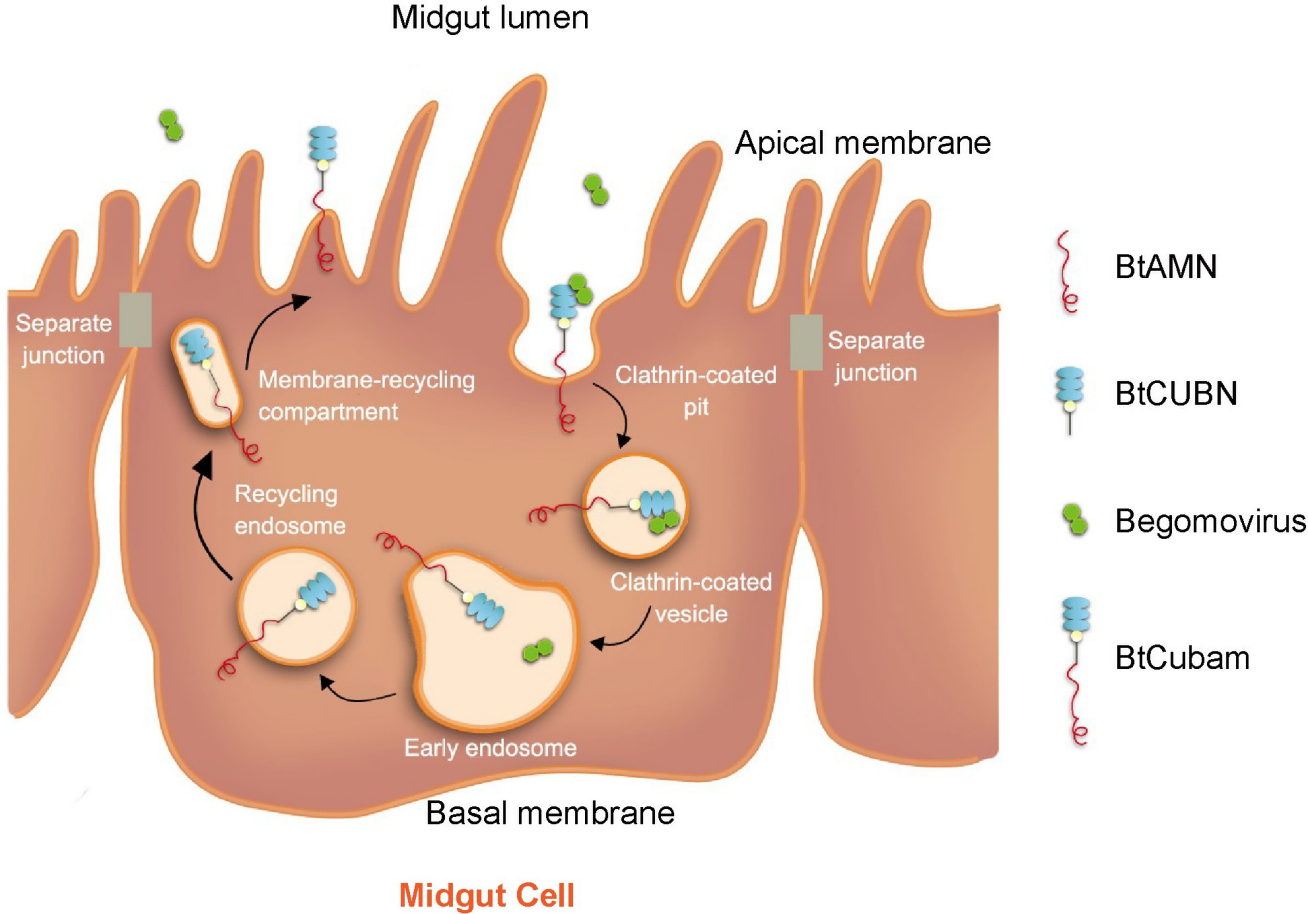
Model of BtCubam-mediated clathrin-dependent endocytosis for begomovirus entry into a midgut cell of the MEAM1 whitefly. Virions may internalize together with BtCubam via its interaction with BtCUBN to cross the apical membrane in the manner of clathrin-dependent endocytosis and then enter the midgut epithelial cells. Next, BtCUBN and virions may dissociate from each other in early endosomes, from where the receptor may recycle to the cell membrane through recycling endosomes.

In the process of circulative virus transmission, specific vector-virus interactions are required for efficient virus acquisition and transport through the body of insect vectors. Based on the findings obtained in this study, we propose a model of BtCubam-mediated endocytosis for begomovirus entry into the midgut cells of the MEAM1 whitefly (Fig 9). BtCUBN and BtAMN form a BtCubam receptor complex in the surface of vector midgut cells. Virions in midgut lumen are firstly captured by the BtCubam complex through the interaction between viral CP and 12–19 CUB domains of BtCUBN, and then virions along with the BtCubam complex are internalized via clathrin-mediated endocytosis. Next, BtCubam receptor complex disassociates from virions in early endosomes and returns to apical membrane via recycling endosomes. Begomovirus may then be transported to the basal membrane of midgut epithelial cells in a process dependent on vesicles induced by Snx12 [26–27].

In mammals, CUBN is recognized as a conserved endocytic receptor with no homology to other endocytic receptors except in the region of EGF repeats, which are also present in receptors of the low-density lipoprotein receptor family [47]. The presence of many CUB domains enable CUBN to bind to various ligands, and multiligand binding properties of CUBN indicate its scavenger receptor function [29,47]. BtCUBN also contains many CUB domains (Fig 1C), which may render the protein vulnerable to exploitation by viruses for the transport through the apical membrane of the MEAM1 whitefly midgut cells via clathrin-dependent endocytosis.

Interestingly, no transmembrane domain was found in CUBN that serves as a peripheral membrane glycoprotein [48]. In earlier studies, the amino-terminal region of CUBN was considered indispensable for anchoring CUBN in the membrane [41]. However, subsequent studies revealed that the transmembrane protein AMN could interact with the EGF domains of CUBN, thereby forming a tightly bound complex with CUBN that serves as a Cubam receptor; AMN is essential for the apical membrane localization and endocytic functions of CUBN [33,35–40]. Our observations show that BtCUBN in combination with BtAMN acts as a receptor complex. These data demonstrate the high evolutionary conservation of CUBN and reveal that CUBN could serve as a potential endocytic receptor for a plant virus, providing novel insights into CUBN biology and plant virus transmission.

Previously, interference in insect acquisition and transmission of virus has been achieved by targeting key factors in insect vectors. For example, inhibiting the expression of *LsST6* led to impaired acquisition and transmission of *Rice stripe virus* by *Laodelphax striatellus*, and blocking the C-lectin in *Aedes aegypti* interrupted *West Nile virus* infection [17,49]. Functional analysis in our study revealed that blocking the interaction between BtCUBN and virus or downregulating the expression of BtAMN reduced competence of the MEAM1 whitefly in begomovirus acquisition and transmission. In addition, CLCuMuV, which is not transmissible by *B*. *tabaci* MEAM1, did not show this interaction, and similarly, TYLCV did not show this interaction in the gut of *T*. *vaporariorum*, a non-vector for TYLCV. These observations indicate that interaction between CUBN and viral CP may be a major portal for begomovirus entry into vector cells. Nevertheless, many questions remain. On the one hand, the key binding sites in viral CP that determine the interaction between CP and BtCUBN remain unclear; on the other hand, according to the released genome of *T*. *vaporariorum*, TvCUBN (Protein number: Tv_01240-RA; 2788 aa) was predicted by chromosome-level genome assembly [50]. However, this sequence of *T*. *vaporariorum* may be incomplete, no signal peptide and EGF repeats were detected by SMART analysis (http://smart.embl-heidelberg.de/). Hence, the divergence between BtCUBN and TvCUBN underlying their differential ability in binding to TYLCV CP warrants further investigation.

Although we have employed in this study a variety of techniques and methods currently available, such as immunofluorescence assay, to examine viral internalization with BtCubam, further evidence of the mediation of viral entry into midgut cells by BtCubam is desirable. For example, temporal dynamics of the crossing of begomovirus over the MEAM1 whitefly midgut cell membrane may be monitored in a time course manner. As yet, this type of monitoring is barely feasible because the crossing of TYLCV over the vector midgut cell *in vivo* is rapid following virus acquisition. Indeed, TYLCV could complete its travel from the vector food channel to haemolymph in a time interval as short as ≤ 2 h [11,31], during the process of which the crossing of cell membrane by virions can be rarely spotted with the methods currently available such as immunofluorescence assay (our own observations). This type of monitoring, however, may become feasible using a MEAM1 whitefly cell line and purified virions. Unfortunately, our repeated efforts on establishing the MEAM1 whitefly cell lines have not yet had any success. The development of a MEAM1 whitefly cell line was reported 20 years ago, but this cell line seems to have lost and no longer available [51, recent personal communication with the authors W.B. Hunter and J.E. Polston]. Czosnek et al. [32] showed that 12–48 h after the initiation of viral acquisition, viruses accumulate in vector midgut, allowing for easy detection of viruses in various stages of travelling. Hence, in our study, following a 48 h period for viral acquisition, whiteflies were used in immunofluorescence analysis for the detection of BtCubam-mediated viral internalization.

In addition, virus entry into vector cells is a complicated multistep process. To hijack vector endocytic machinery, viruses need to first access the cell surface before binding to their receptor(s). Some attachment factors are required to promote virus accumulation on the cell surface, and then following this primary attachment, viruses interact with specific receptor(s) to proceed to internalization, and usually, more than one receptor are required for virus internalization [52, 53]. Our recent work indicates that some potential cellular receptors in the MEAM1 whitefly could be induced by begomovirus infection [54, 55]. Therefore, further efforts are warranted on the identification of attachment factors and additional membrane receptors involved in begomovirus transmission (see S1 Table).

In summary, we have presented evidence suggesting that the internalization of begomovirus virions occur in a BtCubam-mediated, clathrin-dependent manner. Our findings represent a significant step forward in understanding the molecular mechanisms of begomovirus transmission by its insect vector, and provide a basis for developing potential anti-viral transmission strategies in the control of viral diseases via blocking virus traffic within its vector by targeting viral receptors in vectors.

## Materials and methods

### Insects, plants and viruses

The MEAM1 whitefly (mt*COI* GenBank accession number: GQ332577) of the *B*. *tabaci* cryptic species complex and the greenhouse whitefly *T*. *vaporariorum* (mt*COI* GenBank accession number: MH422959) were originally collected from the field and maintained in the laboratory thereafter. the MEAM1 whitefly was reared in insect-proof cages on cotton plants (*Gossypium hirsutum* L. cv. Zhemian 1793) at 26 ± 1°C, 50–70% relative humidity and 14 h light/10 h darkness. The greenhouse whitefly was reared in insect-proof cages on tobacco plants (*Nicotiana tabacum* L. cv. NC89) at 24 ± 1°C, 50–70% relative humidity and 14 h light/10 h darkness. Unless specified otherwise, whiteflies that had emerged within one week were used in all experiments. To obtain virus-infected plants, infectious clones of TYLCV isolate SH2 (GenBank accession number: AM282874) were agro-inoculated into tomato plants (*Solanum lycopersicum* L. cv. Hezuo903) at the 2–3 true leaf stage; TYLCCNV isolate Y10 (GenBank accession number: AJ319675) with its conjugated beta-satellite (GenBank accession number: AJ421621) were agro-inoculated into 2–3 true leaf stage tobacco plants; clones of CLCuMuV isolate GD37 (GenBank accession number: JN968573) with its conjugated beta-satellite (GenBank accession number: JN968574) were agro-inoculated into 2 true leaf stage cotton plants (*Gossypium hirsutum* L. cv. Xinhai 21). Cotton plants of cv. Zhemian 1793 could not be infected by CLCuMuV through either the MEAM1 whitefly transmission or agroinoculation, whereas those of cv. Xinhai 21 could be readily infected. Approximately four weeks post inoculation, the status of infection was determined by visual inspection of symptoms and PCR detection, and then used for experiments. Primers are listed in S2 Table.

### Antibodies

TYLCV CP, TYLCCNV CP and CLCuMuV CP were detected using anti-TYLCV CP mouse monoclonal antibody (mAb), which was kindly provided by Professor Jian-Xiang Wu (Institute of Bio-technology, Zhejiang University). Mouse polyclonal antibodies (pAbs) of BtAMN and BtCUBN were produced by HuaBio (China), and 26-349aa of BtAMN and 3593-3847aa of BtCUBN fusion with His tag expressed in prokaryotic cells were used as immunogens respectively, and primers are listed in S2 Table. Rabbit pAbs of BtCUBN_762-775aa_ and BtCUBN_1938-1951aa_ were produced by HuaBio (China) using synthetic peptides conjugated to KLH, and the peptide sequences were CSPKHPEKNDEYVSD and CHSGSQADTTGKGFT respectively. Other antibodies used were anti-CUBN rabbit mAb (Cat. No. ab191073; Abcam), anti-β-Actin mouse mAb (Cat. No. E021020-01; Earthox); anti-clathrin heavy chian sheep pAb (Cat. No. ab223272; Abcam), anti-GST mAb antibody (Cat. No. 2624; CST), anti-HA mouse mAb antibody (Cat. No. ab18181; Abcam), anti-His mouse mAb (Cat. No. A00186; Genescript), donkey anti-goat IgG HRP (Cat. No. A0181; Beyotime), goat anti-rabbit IgG HRP (Cat. No. E030120; Earthox), goat anti-mouse IgG HRP (Cat. No. E030110; Earthox), goat anti-rabbit IgG Alex Flour 647 (Cat. No. A21245; Invitrogen), goat anti-mouse IgG Alex Flour 549 (Cat. No. E032310-02; Earthox), donkey anti-mouse IgG Alex Flour 647 (Cat. No. ab150111; Abcam), donkey anti-rabbit IgG Alex Flour 488 (Cat. No. ab150073; Abcam), donkey anti-sheep IgG Alex Flour Cy3 (Cat. No. A0502; Beyotime), nanogold goat anti rabbit IgG (Cat. No. 2003; Naboprobes). Phalloidine (Cat. No. P5282; Sigma) was used to label the midgut apical membrane. Rab 5 and Rab11 rabbit pAb were chosen according to Xia et al. [27]. All of these commercial antibodies or antibodies produced in our study were detected by western blot analysis (S7 and S8 Figs).

### GST pull-down

The DNA region coding for the TYLCV CP was cloned into pGEX-6p-1 for the expression of GST- CP. This recombinant protein was expressed in *E*.*coli* strain Rosetta and then purified. To obtain the fusion proteins of His-BtAMN and HA-BtAMN, gene of *BtAMN* was synthesized and then cloned into pFastBac HTB vector or the modified pFastBac HTB vector in which the His tag was replaced by Ha tag via clonexpress II one step cloning kit (Cat. No. C112-01; Vazyme). And different regions of *BtCUBN* were synthesized and then cloned into pFastBac HTB vector for the expression of the His fusion proteins. These recombinant proteins were expressed in Sf 9 cells using Bac-to-Bac TOPO expression system (Cat. No. A11101; Invitrogen). GST-CP or GST was bound to glutathione agarose beads (Cat. No. 17-5132-01; GE Healthcare) for 2–4 h at 4°C. Then the mixtures were centrifuged for 5 min at 1000 rpm and the supernatants were discarded. These agarose beads were washed five times with 1×PBS. The total native proteins of Sf 9 cells, in which BtAMN or different regions of BtCUBN were expressed, were extracted by minute total protein extraction kit (Cat. No. SN-002; Invent), and native proteins of the MEAM1 whitefly or the greenhouse whitefly were extracted by cytoplasmic extraction buffer (Cat. No. SC-003; Invent). Then, these prey proteins were mixed with the washed beads and incubated for 4 h at 4°C respectively. Next, these mixtures were centrifuged and washed five times with 1×PBS, and then the bead-bound proteins were eluted by boiling in PAGE buffer (Cat. No. FD002; FDbio) for 10 min. Finally, SDS-PAGE electrophoresis was conducted, and coomassie blue (Cat. No. B105004; Aladdin) or western blot was used to detect the GST-CP affinity captures. Primers are listed in S2 Table.

### LC-MS/MS analysis

We used GST-TYLCV CP as bait protein, and native proteins of the MEAM1 whitefly extracted by cytoplasmic extraction buffer (Cat. No. SC-003; Invent) as prey protein for GST pull-down. Next, proteins were recovered from SDS-PAGE gels and subjected to LC–MS/MS assay (Fig 1B). LC-MS/MS was performed following the methods described by Huang et al. [56]. Briefly, three steps were performed, namely (1) peptide digestion; (2) LC-MS/MS analysis; and (3) peptides identification by searching the MEAM1 protein sequence (http://www.whiteflygenomics.org).

### Gene transcription analysis

For the analysis of *BtCUBN* transcription in the MEAM1 whitefly midgut and carcass (whole body sans-gut), female adults were collected and dissected for analysis. From each of the three replicates, 100 midguts and 30 carcasses were collected, respectively. RNA extraction was done using TRIzol (Cat. No.15596018; Invitrogen), and reverse transcription was performed using PrimeScript RT reagent Kit (Cat. No. RR047A; Takara) as per manufacturer’s protocol. qPCR was performed on CFX96 Real-Time PCR Detection System (Bio-Rad, USA) with SYBR Premix Ex Taq II (TaKaRa, Japan). All primers for qPCR analysis are listed in S2 Table.

### Immunogold electron microscopy (IEM)

Midguts of viruliferous MEAM1 female adults were dissected, and then IEM was performed as follows: (1) fixed overnight at 4°C with 4% PFA and 0.15% glutaradehyde (Cat. No. A500484; Sangon Biotech) in 0.1 M PB, PH = 7.4; (2) discard fixative and washed in 0.1 M PB for three times; (3) incubated with 50 mM glycine in 0.1M PB for 30 min and washed in 0.1 M PB three times; (4) permeabilized using 0.05% Triton X-100 in 0.1M PB for 30 min, washed in 0.1 M PB three times, and then blocked using 0.1% BSA-cTM (Cat. No. 25558; Electron Microscopy Sciences) in 0.1 M PB, PH = 7.4 for 30 min; (5) incubated with primary antibodies overnight at 4°C, and washed in blocking buffer six times; (6) incubated with secondary antibodies overnight at 4°C, and washed in blocking buffer six times; (7) fixed with 2.5% GA for 2 h at room temperature, washed in 0.1MPB three times, and then washed in ddH_2_O six times; (8) washed in 0.02M sodium citrate buffer three times, incubated with HQ silver enhancement kit (Cat. No. 2012; Naroprobes) for 2 min and washed in ddH_2_O six times, and then incubated with ddH_2_O overnight at 4°C; (9) washed in 0.1M PB three times and incubated with 1% OsO4 in 0.1M PB at 4°C for 30 min; (10) washed in ddH_2_O three times, incubated with 2% uranyl acetate in ddH_2_O for 30min, and then washed in ddH_2_O three times again; (11) dehydrated using ethanol of different concentration (50%, 70%, 90%) and acetone, and incubate with each reagent for 20 min; (12) embedded the specimens in Epon and sectioned using a LEICA EM UC7 ultramicrotome, and sections were observed using TecnaiG2 Spirit120kVcryo-EM.

### Immunofluorescence

Midguts of female whitefly adults were dissected and immunofluorescence was performed using the following procedures: (1) midguts were firstly incubated for 2 h in 4% paraformaldehyde (PFA, Cat. No. F0001; MultiSciences); (2) 4% paraformaldehyde was removed and midguts were washed in 1×PBS three times; (3) samples were then blocked and permeabilized using 0.4% Triton X-100 in 3% BSA (Cat. No. A3828; MultiSciences) for 2 h; (4) primary antibody was added and the mixture was incubated for 2 h at room temperature or overnight at 4°C; (5) the primary antibody was removed and midguts were washed in 1×PBS three times, and then incubated with secondary antibody or fluorescence-labeled phalloidine for 2 h at room temperature; (6) the nucleus were stained with 100 nM 4′,6-diamidino-2phenylindole (DAPI) (Cat. No. ab104139; Abcam) for 2 min at room temperature. All specimens were viewed under LSM 780 (ZEISS, Germany). The actual testing procedures varied for different experiments and the details for each experiment are presented in the legend of a diagram for a given experiment. We conducted plot profile to help analyze co-localization (avoid making judgments only via visual sense): import the JPG format image (single channel) into image J software → select the whole image (ctrl+A) → analyze → plot profile → export data → generate the plot graph by GraphPad Prim 7. Fluorescence intensity analysis was also conducted by Image J software. Each of the immunofluorescence images is typical of at least two independent experiments with multiple samples.

### Transmission electron microscopy (TEM)

Midguts of female adults of the MEAM1 whitefly were dissected, fixed overnight at 4°C with 2.5% glutaraldehyde in phosphate buffer (0.1M, pH = 7.0), and washed three times with 1×PBS; then post-fixed with 1% OsO4 in phosphate buffer for 1.5 h, washed three times with 1×PBS and dehydrated in a graded series of ethanol (30%, 50%, 70%, 80%, 90%, 95% and 100%), and finally embedded in Spurr resin. The specimen was sectioned using a LEICA EM UC7 Ultramicrotome. Sections were stained using uranyl acetate and alkaline lead citrate for 5 to 10 min respectively, and then observed under a Hitachi Model H-7650 TEM.

### Co-immunoprecipitation (Co-IP) Assay

After feeding on virus-infected tomato plants for a 7 d acquisition access period (AAP), the MEAM1 whitefly or the greenhouse whitefly was collected and native proteins were extracted using cytoplasmic extraction buffer (Cat. No. SC-003; Invent), and then incubated with anti-TYLCV CP mouse mAb overnight at 4°C. Pre-immune sera (Cat. No. A7028; Beyotime) was used as control. After the incubation, protein G-Sepharose beads (Cat. No. 17-0405-03; GE Healthcare) were added to the mixture and further incubated at 4°C for 2–4 h. Next, the mixtures were washed five times using 1×PBS, and then the co-immunoprecipitated proteins were eluted by boiling in PAGE buffer for 10 min. Finally, SDS-PAGE electrophoresis was conducted and beads-bound proteins were detected by western blot using anti-CUBN rabbit mAb. In this study, we used SurePAGE, Bis-Tris, 4–20% gels (Cat. No. M00656; GenScript) and 1×MOPS running buffer (Cat. No M00680-500; GenScript) for western blot analysis.

### Purification of TYLCV virions

Firstly, 3–4 true leaf stage *Nicotiana benthamiana* plants were agro-inoculated with TYLCV isolate SH2. Three-four weeks later, virions were purified from leaves showing typical symptom using method II as described by Czosnek et al. [57], omitting the step of sucrose gradient [58]. Pellets containing virions were re-suspended in 0.1 M/L phosphate buffer containing 0.2 mM/L EDTA with pH at 7.0 and the solution was stored in a -80°C freezer after the addition of equal volume of 30% glycerol. The copy number of stored TYLCV virions was 1.06×10^10^. This copy number was estimated as normalized to a standard curve, which was made by qPCR reaction using the serial dilutions of plasmid extracted from the infectious clone of TYLCV as the template.

### Membrane feeding

The MEAM1 adults were collected and released into glass tubes with a diameter of 1.5 cm and a length of 10 cm. One opening of the tube was covered with double layers of parafilm filled with a diet solution and the other was covered with gauze [25]. Anti-CUBN rabbit mAb was added into 15% sucrose solution with a dilution of 1:50 (final concentration: 14.24 μg/ml), and rabbit pre-immune serum (Cat. No. A7016; Beyotime) with the same concentration was used as control. The duration of membrane feeding was 24 h. To enable simultaneous acquisition of both antibody and virions by the MEAM1 whitefly, anti-CUBN rabbit mAb (1:50) was added to 30% sucrose solution mixed with equal volume of TYLCV virions (viral copy number 5.0×10^9^). The duration of membrane feeding was 48 h, the “fodder” was renewed once a day.

### ds*BtAMN* synthesis and microinjection

Specific primers containing T7 polymerase promoter sequences were used in PCR to amplify templates which were used for dsRNA synthesis by using T7 high yield RNA transcription kit (Cat. No. TR101-01; Vazyme). Then, the dsRNA was purified using phenol: chloroform extraction and isopropanol precipitation, and then the purified dsRNA was re-suspended in RNA nuclease-free water. Prior to use, the quality and concentration of dsRNA was examined using agarose gel electrophoresis and Nanodrop (Thermo Scientific, USA). Primers used for DNA template synthesis are listed in S2 Table. Microinjection was performed using capillary and FemtoJet (Eppendorf, Germany), and approximately 6 nl of purified dsRNA (3 μg/μl) was injected into the thorax of each of the female MEAM1 whitefly adults. DsRNA targeting *GFP* was used as control. After injection, insects were reared on cotton leaves for two days for recovery, and then used for viral acquisition. Efficiency of dsRNA mediated gene silencing was verified using qPCR, western blot and immunofluorescence. The MEAM1 whitefly adults were collected as groups of 30, and three replicates were conducted for RNA isolation, cDNA synthesis, and qPCR analysis. For western blot analysis, the MEAM1 whitefly adults were collected as one group of 100, and protein extraction (Cat. No. P0013B; Beyotime) and then western-blot were conducted. BCA protein assay (Cat. No. 23250; Thermo Scientific) was used to determine the concentration of protein samples, and total protein in western-blot analysis was controlled at the same amount for ds*GFP* and ds*BtAMN* treatment. For immunofluorescence analysis 6–8 midguts of females were used.

### Detection of virus in the MEAM1 whitefly

Post antibody feeding or dsRNA injection, the MEAM1 adults from the two treatments were subjected to oral virus acquisition on two symmetrical leaves of the same height on the same branch for 24 h when tomato plants were used as source of inoculum. When tobacco plants were used as source of inoculum adults of the two treatments were subjected to oral viral acquisition on the two sides of a symmetrical leaf. Then, the adults were collected as one group of 100 to analyze the protein level of TYLCV CP in the MEAM1 whitefly whole body using western blot as described above. For qPCR detection of virus in the MEAM1 whitefly whole body, female adults were collected individually. As for organs, midgut and salivary glands were dissected and haemolymph was collected from one female and treated as one sample. The haemolymph was collected from whitefly abdomen using a capillary with a fine point of ~1 μl in volume. For each treatment, 16–34 samples were collected. These samples were then subjected to DNA extraction using lysis buffer as described previously [25] and qPCR was conducted as described above with primers listed in S2 Table.

### Viral transmission by the MEAM1 whitefly

Post antibody feeding or dsRNA injection, the MEAM1 adults were allowed to feed on virus-infected tomato plants for 24 h, and then the females were transferred to feed for 48 h on leaves of un-infected tomato/tobacco seedlings using clip-cages, with two females in each cage. For the MEAM1 whitefly acquiring both antibody and TYLCV virions, groups of six females each were transferred to feed for 48 h on leaves of un-infected tomato seedlings using clip-cages. The females were then removed, and the plants were sprayed with imidacloprid at a concentration of 20 mg/L to kill all the eggs on the leaves. Thirty days later, the top fully-expanded leaf of each test plant was harvested for DNA extraction and PCR detection of virus. Primers are listed in S2 Table. For each treatment, three replicates were conducted with each consisting of 6–15 test plants.

### Statistical analysis

All qPCR data were calculated using 2^-ΔCt^ as normalized to *β-Actin*; all proportional data were transformed by arcsine square root for statistical analysis, and were back-transformed to proportions for presentation. Independent t-test was used in this study and the differences between treatments were considered significant when *P<0.05; **P<0.01, ***P<0.001. All the statistical analyses were performed with SPSS 20.0 (SPSS Inc., USA).

## Supporting information

S1 Fig The MEAM1 whitefly midgut structure presented by TEM (A) and immunofluorescence (B). The arrows indicate the order of viral movement. Midgut apical membrane was stained by phalloidine conjugated to Alex Flour 488 (orange); cell nucleus was stained by DAPI (Alex Flour 405, blue); BtCUBN was labelled with Alex Flour 647 (green). ML: midgut lumen; MV: microvilli; C: cytoplasm; N: nucleus; BM: basal membrane.
(TIF)

S2 Fig The structure, expression and distribution of BtAMN in the MEAM1 whitefly midgut. (A) Schematic representation of BtAMN structure. (B) *BtAMN* transcription levels in the MEAM1 whitefly midgut and carcass. Midguts of females were removed and the remains were collected as carcass. The MEAM1. Data were presented as mean ± SEM, n = 3 (Independent t-test, *P < 0.05). (C) Immunofluorescence detection of BtAMN in the MEAM1 whitefly midgut cells. the MEAM1 adults were allowed to feed on TYLCV-infected plants for a 48 h AAP and used in immunofluorescence assay. Midgut apical membrane was stained by phalloidine conjugated to Alex Flour 488 (orange); cell nucleus was stained by DAPI (Alex Flour 405, blue); BtAMN was labelled with Alex Flour 549 (red). Scale bar, 5μm. ML: midgut lumen; MV: microvilli; N: nucleus; C: cytoplasm; BM: basal membrane.
(TIF)

S3 Fig BtAMN exhibited no binding activity to TYLCV CP. BtAMN was expressed in fusion with His tag (A-B) or HA tag (C-D) in Sf 9 cells (prey protein); purified GST or GST-TYLCV CP was used as bait protein. After GST pull-down analysis, the prey protein was detected by anti-His (A) mouse mAb or anti-BtAMN rabbit pAb (B and D) or anti-HA mouse mAb antibody (C) respectively; bait protein was detected by anti-GST mouse mAb. His-BtAMN, ≈ 54 kDa; HA-BtAMN, ≈ 54 kDa; GST, ≈ 26 kDa; GST-TYLCV CP, ≈ 54 kDa.
(TIF)

S4 Fig Involvement of BtCubam in TYLCCNV infection of the MEAM1 whitefly. The MEAM1 adults were allowed to feed on TYLCCNV-infected plants for a 48 h AAP for immunofluorescence analysis. (A) Co-localization among TYLCCNV CP, BtCUBN and clathrin. TYLCCNV CP was labelled with Alex Flour 647 (red); BtCUBN was labelled by its rabbit mAb (Alex Flour 488, green); clathrin was labelled with Alex Flour 549 (orange) and cell nucleus was stained by DAPI (Alex Flour 405, blue). Scale bar, 5μm. (B-C) Following anti-CUBN rabbit mAb feeding (B) or ds*BtAMN* interference (C), the MEAM1 whitefly adults were transferred to TYLCCNV-infected plants for a 24 h AAP for qPCR analysis: quantity of virus in whitefly whole body (WB) and different organs: midgut (MG), haemolymph (HL) and salivary gland (SG). (D-E) Following anti-CUBN rabbit mAb feeding (D) or ds*BtAMN* interference (E), the MEAM1 whitefly adults were transferred to TYLCCNV-infected plants for a 24 h AAP, then, twofemales were collected and virus transmission was performed, data shown are mean ± SEM, n = 3. Statistically significant differences are indicated as *P < 0.05; **P < 0.01, ***P < 0.001 (Independent t-test). The plot profile presented was used to help verify the visual co-localization.
(TIF)

S5 Fig No interaction between CLCuMuV CP and BtCUBN. The MEAM1 whitefly was allowed to feed on CLCuMuV-infected cotton for a 7 d AAP for immunofluorescence analysis. Anti-CUBN rabbit mAb was used here. (A-B) No interaction was found between CLCuMuV CP and BtCUBN via GST pull-down (A) and Co-IP (B) analysis. TvCUBN, ≈ 423 kDa; GST, ≈ 26 kDa; GST-TYLCV CP, ≈ 54 kDa; light chain, ≈ 26 kDa; heavy chain, ≈ 55 kDa. (C) Detection of viral signals in the midgut of the MEAM1 whitefly. (D) No co-localization between extracellular CLCuMuV CP and BtCUBN was observed. For C-D, midgut apical membrane was stained by phalloidine (Alex Flour 488, orange); cell nucleus was stained by DAPI (Alex Flour 405, blue); BtCUBN was labelled with Alex Flour 647 (green); viruses were detected by Alex Flour 549 (red). Scale bar, 5μm. The plot profile presented was used to help verify the visual co-localization.
(TIF)

S6 Fig No interaction between TYLCV CP and TvCUBN. The greenhouse whitefly was allowed to feed on TYLCV-infected plants for a 7 d AAP for immunofluorescence analysis. Anti-CUBN rabbit mAb was used here. (A-B) No interaction was found between TYLCV CP and TvCUBN via GST pull-down (A) and Co-IP (B) analysis. TvCUBN, > 250 kDa; GST, ≈ 26 kDa; GST-TYLCV CP, ≈ 54 kDa; light chain, ≈ 26 kDa; heavy chain, ≈ 55kDa. (C) The greenhouse whitefly midgut structure presented by immunofluorescence. ML: midgut lumen; MV: microvilli; C: cytoplasm; N: nucleus; BM: basal membrane. (D) The distribution of virions in the midgut of the greenhouse whitefly. (E) No co-localization between extracellular TYLCV CP and TvCUBN was observed. In C-E, midgut apical membrane was stained by phalloidine (Alex Flour 488, orange); cell nucleus was stained by DAPI (Alex Flour 405, blue); TvCUBN was labelled with Alex Flour 647 (green), viruses were detected by Alex Flour 549 (red). Scale bar, 5μm. The plot profile presented was used to help verify the visual co-localization.
(TIF)

S7 Fig Western blot analysis of commercial antibodies used in this study. (A) the MEAM1 whitefly (a) or the greenhouse whitefly (b) proteins extracted using cytoplasmic extraction buffer (Cat. No. SC-003; Invent) were detected using anti-CUBN rabbit mAb. (B) His-BtAMN expressed in Sf 9 cells was detected using anti-His mouse mAb. (C) HA-BtAMN expressed in sf 9 cells was detected using anti-HA mouse mAb. (D-E) the MEAM1 whitefly total proteins extracted by PIPA lysis buffer (Cat. No. SN-002; Beyotime) were detected using anti-clathrin heavy chain sheep pAb (D) and anti-β-Actin mouse mAb (E) respectively. (F) Purified GST, GST-TYLCV CP and GST-CLCuMuV CP following by prokaryotic expression system were detected using anti-GST mouse mAb. Protein extraction of Sf 9 cells were isolated by minute total protein extraction kit (Cat. No. SN-002; Invent).
(TIF)

S8 Fig Western blot analysis of antibodies produced in this study. (A) His-BtCUBN_1-865aa_ or His-BtCUBN_514-1336aa_ expressed in sf 9 cells was detected using anti-BtCUBN_762-775aa_ rabbit pAb. (B) His-BtCUBN_1104-2088aa_ (a) or His-BtCUBN_1945-3092aa_ (b) expressed in sf 9 cells was detected using anti-BtCUBN_1938-1951aa_ rabbit pAb. (C) His-BtCUBN_2857-3827aa_ expressed in sf 9 cells (a) or the MEAM1 whitefly proteins (b) extracted by cytoplasmic extraction buffer (Cat. No. SC-003; Invent) were detected using anti-BtCUBN_3593-3847aa_ mouse pAb. (D) the MEAM1 whitefly proteins (a) extracted by cytoplasmic extraction buffer (Cat. No. SC-003; Invent) and His-BtAMN (b) or HA-BtAMN (c) expressed in sf 9 cells were detected using anti-BtAMN mouse pAb. (E) Total proteins of viruliferous the MEAM1 whitefly or the greenhouse whitefly were extracted by PIPA lysis buffer (Cat. No. SN-002; Beyotime) and then detected using anti-TYLCV CP mouse mAb. Protein extraction of Sf 9 cells were isolated by minute total protein extraction kit (Cat. No. SN-002; Invent).
(TIF)

S9 Fig Images for immunofluorescence detection of BtAMN in the midguts of control and silenced MEAM1 whitefly. BtAMN was labelled with Alexa Fluor 549 (red). Cell nucleus was stained with DAPI (Alexa Fluor 405, blue). Scale bar, 50 μm.
(TIF)

S1 Table The MEAM1 whitefly Proteins identified using GST pull-down followed by LC-MS/MS analysis. (XLSX)

S2 Table Primers used in this study. (XLSX)
